# Dietary and Nutraceutical Interventions as an Adjunct to Non-Surgical Periodontal Therapy—A Systematic Review

**DOI:** 10.3390/nu15061538

**Published:** 2023-03-22

**Authors:** Johan Peter Woelber, Katharina Reichenbächer, Tara Groß, Kirstin Vach, Petra Ratka-Krüger, Valentin Bartha

**Affiliations:** 1 Department for Operative Dentistry and Periodontology, Faculty of Medicine, University of Freiburg, Hugstetter Str. 75, 79106 Freiburg, Germany; 2 Institute of Medical Biometry and Statistics, Faculty of Medicine, University of Freiburg, Zinkmattenstr. 6A, 79108 Freiburg, Germany; 3 Department for Conservative Dentistry, University of Heidelberg, Im Neuenheimer Feld 400, 69120 Heidelberg, Germany

**Keywords:** nutrition, diet, diet intervention, non-surgical periodontal therapy

## Abstract

The aim of this study was to conduct a systematic literature review on the influence of dietary and nutraceutical interventions as an adjunct to non-surgical periodontal therapy (NSPT). A literature search for randomized, controlled clinical trials (RCTs) was performed in PubMed, the Cochrane Library, and the Web of Science. Trial inclusion criteria included the application of a defined nutritional intervention (food, beverages, or supplements) adjunctive to NSPT compared to NSPT alone with at least one measured periodontal parameter (pocket probing depths (PPD) or clinical attachment level (CAL)). Of 462 search results, 20 clinical trials relating to periodontitis and nutritional interventions were identified, of which, in total, 14 studies could be included. Eleven studies examined supplements containing lycopene, folate, chicory extract, juice powder, micronutrients and plant extracts, omega-3 fatty acids, vitamin E, or vitamin D. Three studies examined food-based interventions (kiwifruit, green or oolong tea). Due to limited information on within-group differences in the studies, results were descriptively analyzed. A significant positive effect on periodontal parameters (PPD, bleeding on probing) was found for vitamin E, chicory extract, juice powder, green tea, and oolong tea. Heterogeneous effects were found for lycopene, folate, omega-3 fatty acids, and vitamin D. No effects on PPD were found for adjunct kiwifruit (in combination with NSPT). Risk of bias via RoB2 revealed a low risk of bias with some concerns. There was a high heterogeneity in the type of nutritional interventions. The adjunctive use of various supplements and green/oolong tea led to positive and significant effects of the nutritional interventions on clinical periodontal outcome parameters. In the context of non-surgical periodontal therapy, an adjunctive intake of micronutrients, omega-3 fatty acids, green/oolong tea, and polyphenols and flavonoids could be beneficial. Long-term clinical studies with full data reports (especially within-group differences) are needed for conducting a meta-analysis.

## 1. Introduction

Periodontitis is a multifactorial inflammatory disease leading to tissue breakdown and subsequent tooth loss [1]. It develops due microbial and host factors that influence inflammation and can be described as an immuno-inflammatory disease, determined by the interaction between bacterial colonization and host immunology [2]. Genome-wide association studies throughout the last decade underlined the complex genetic nature of periodontitis [3,4]. Apart from this, there are several modifiable risk factors such as stress, tobacco use, and other environmental factors [5]. These factors are important additional targets for prevention and treatment strategies. The current European Federation of Periodontology (EFP) guidelines recommend improving metabolic control of diabetes and smoking cessation [6]. On the other hand, the role of physical activity, weight reduction, and dietary counselling remains unclear, and the EFP guidelines call for further evidence on these factors.

Nutritional interventions have been shown to be important therapeutic approaches in the treatment of non-communicable diseases such as diabetes mellitus, cardiovascular diseases, and cancer [7,8,9]. Regarding inflammatory diseases, Mediterranean, Nordic, and Paleo diets as well as modern dietary styles that are low in consumption of processed foods, refined sugars or fats showed anti-inflammatory effects [10,11,12]. In contrast, the Western diet predominantly consists of highly processed and micronutrient-poor foods, based on processed carbohydrates like sugar and starch, which are low or even free of dietary fibers and contain pro-inflammatory fats, like saturated and trans-fatty acids. Thus, it has been associated with numerous diseases, including diabetes, hypertension, and cardiovascular disease [13,14].

Regarding oral inflammatory diseases, anti-inflammatory dietary interventions low in carbohydrates and rich in omega-3 fatty acids, vitamins C and D, antioxidants, and dietary fiber have been shown to decrease periodontal inflammatory parameters [15,16,17,18,19]. Taking into consideration the effects of nutrition on inflammatory diseases, dietary strategies may support periodontal therapy alone or in combination with subgingival instrumentation. Recently, some association and cross-sectional studies have demonstrated that high micronutrient and fiber [20], Mediterranean or Dietary Approach to Stop Hypertension [21] dietary patterns are associated with a lower risk for periodontal disease. Furthermore, a number of randomized controlled trials investigating nutritional interventions in context of periodontitis and periodontal therapy were conducted. However, to the best of the authors’ knowledge, there is no summarizing evidence yet in the form of a systematic review. Therefore, this systematic review aimed to examine the current evidence on nutritional interventions as an adjunct to NSPT.

## 2. Materials and Methods

### 2.1. Eligibility Criteria

The study was performed according to the PRISMA protocol (Preferred Reporting Items for Systematic Reviews and Meta-Analysis, see ([22], Appendix 1)) and registered under PROSPERO (https://www.crd.york.ac.uk/prospero/; accessed on 28 April 2020), with the registration number CRD42020139683.

Following basic research question was formulated: “What impact do nutraceuticals have as an adjunct to non-surgical periodontal therapy in periodontitis patients?”

This research question was investigated using the population, intervention, control, and outcomes (PICO) format: The diet of patients who were generally healthy but had periodontitis (population) required modification by supplementation or dietary change (intervention) as an adjunct to non-surgical periodontal therapy. The comparison had to consist of a control group that did not experience a nutritional intervention or received a placebo. The aim (outcome of interest) of the study was to determine if such an intervention had a significant influence on pocket probing depths (PPD, primary outcome), clinical attachment loss (CAL), percentage of sites with bleeding on probing (BOP), total antioxidant capacity, and serum lipid levels and cytokines in serum and saliva (secondary outcomes). The literature search aimed to find randomized clinical trials (RCTs) of nutritional interventions in subjects with periodontitis. The target questions, search term, inclusion and exclusion criteria were formulated in a PROSPERO protocol.

The included studies had to fulfill the following criteria:

- RCT;
- Blinding of the participants and/or examiners;
- Generally healthy subjects with periodontitis;
- Periodontal examination with at least PPD;
- An intervention within the study through a modified, defined diet regarding its containing food compilation or administration of supplements/nutraceutical, used as an adjunct to non-surgical periodontal therapy;
- Follow-up period of at least 2 months.

The exclusion criteria were as follows:

- Studies examining other forms of periodontitis, such as periodontic-endodontic lesions or apical periodontitis as a main research question;
- Studies applying nutraceuticals on the periodontium only locally;
- Studies that included participants under conditions like pregnancy or breastfeeding;
- Studies with participants who were on a combined intake of medications with the nutritional intervention;
- Use of adjuncts like antibiotics or antiseptics;
- Other study designs, such as case series, cohort studies, or animal studies.

### 2.2. Search Strategy

The literature search included all articles published from 1 January 1945 to 26 May 2022 in three databases: Medline via PubMed, the Cochrane Library (Cochrane central), and the Web of Science (Thomson Reuters).

The results were managed and checked for duplicates using the literature management program Zotero (zotero.org, Corporation of Digital Scholarship, Vienna, VA, USA). A single search term was formed for all three literature databases. To describe the type of intervention, the search terms “diet” and “nutrition” were chosen. These were linked with the Boolean operator “OR”. The terms “periodontitis” and “periodontal” were used to describe the searched subjects and their disease; they were also linked with the Boolean operator “OR”. To limit the search on periodontal therapy, the term “therapy” was added. All search terms, “diet” OR “nutrition” and “periodontitis” OR “periodontal” and “therapy”, were again linked with the Boolean operator “AND”. Thus, the final search term was “(diet OR nutrition OR antioxidant OR vitamin OR fruit OR fatty acids) AND (periodontitis OR periodontal) AND therapy”. If the full texts could not be determined or if the literature was “gray literature”, Google Scholar (Google LLC, Mountain View, CA, USA) was additionally used. After the database search, a manual search was performed. A hand-search was done in the following preselected journals: The Journal of Periodontology (from 1931), The Journal of Clinical Periodontology (from 1974), and The Journal of Periodontal Research (from 1966). Additionally, a backward search was done after the screening process, checking the reference lists of all at that point included publications for cross-references.

The literature search and evaluation were performed independently by two authors who were blinded to each other (KR, TG). The second independent investigator (TG) performed a second rating by using the defined search terms. For this, all three databases were searched and evaluated again. Possible disagreements in search results were resolved by discussion with a third author (JPW). Initially, titles as well as abstracts were read and assessed using the inclusion and exclusion criteria. For these initially included studies, the full text was also searched, assessed against the established criteria, and included or excluded accordingly.

### 2.3. Risk of Bias of the Included Studies

To achieve the highest possible quality and general validity, risk of bias was assessed by the Cochrane risk of bias tool version 2 (RoB2), based on five different domains regarding randomization, intervention deviations, missing outcome data report, measurement of outcome, outcome data selection, and overall risk of bias resulting from the ranking of the five domains. All risk of bias domains could be classified as low risk, some concerns, or high risk. The outcome of RoB2 was visualized via robvis [23].

### 2.4. Quality of Evidence

Quality of evidence assessment was performed using the Grading of Recommendations Assessment, Development and Evaluation (GRADE) according to Schünemann 2009. It includes the study design, the study limitations, inconsistencies, indirectnesses, imprecisions, and number of included patients per study group to grade each treatment outcome. Results were taken into a table presenting the summary of findings rating the quality of evidence between very low, low, moderate, or high [24]. 

### 2.5. Meta-Analysis

In order to perform a meta-analysis, all included studies were examined for weighted mean differences and standard errors. In case of missing data, the corresponding authors were contacted via email.

## 3. Results

### 3.1. Included Studies

**Figure 1:**
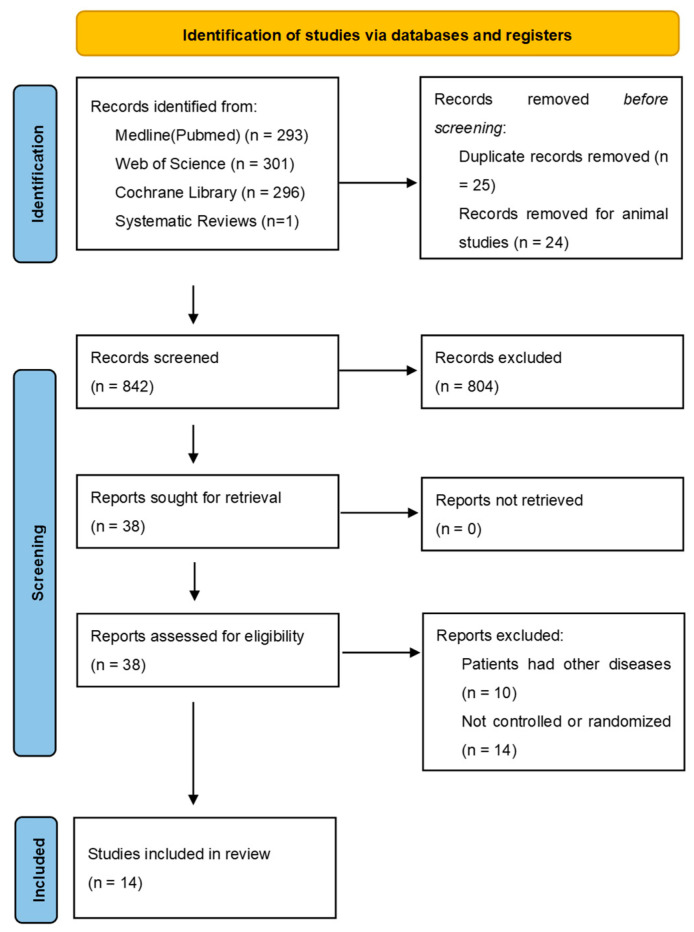
PRISMA 2020 flow diagram; systematic literature search in PubMed, Web of Science, and Cochrane Library with the defined search term and by hand search/gray search.

Within the initial search, 890 articles were found on PubMed, Web of Science, and in the Cochrane Library (Figure 1). Before screening, 25 duplicates and 24 animal studies were removed. After reviewing 841 titles and abstracts, 37 clinical trials related to periodontitis, non-surgical periodontal therapy, and nutritional interventions remained. Further full text analyses led to the exclusion of 24 studies, and 14 studies were included in the systematic review.

Excluded studies according to full text analysis are reported in Supplementary Table S1.

Regarding the second rating, the investigator’s agreement was 99.9% (*p* = 0.999) with a Cohen’s Kappa coefficient of 0.87 [25].

### 3.2. Characteristics of the Included Studies

All 14 studies were homogenous in their design as randomized controlled trials with blinded examiners, conducted in a parallel group design. The type of intervention showed high heterogeneity:

- Daily intake of 2 mg lycopene capsules with additional micronutrients over 2 months [26].
- Chicory extract capsules over a period of 2 months [27].
- Folic acid capsules over a period of 6 months [28].
- Daily consumption of oolong tea for 30 days [29].
- Fruit–vegetable or fruit–vegetable–berry capsules given over a period of 8 months [30].
- Four interventions examined the effect of omega-3 fatty acid capsules [31,32,33,34]. The daily intake ranged from approximately 50 mg [32] to 4400 mg [34] over a period of either 12 weeks [31,34], 6 months [32], or 1 year [33].
- Two studies reported on the effects of vitamin D supplementation [35,36]. Perić et al. [36] examined the effect of 25,000 IU Vitamin D3 per week over 6 months, and Gao et al. [35] studied the effects of daily intake of either 2000 IU or 1000 IU Vitamin D3 over 3 months.
- The daily intake of fruits was examined in one study [37]. The intervention of Graziani et al. [37] included the daily consumption of two kiwifruits over a period of 5 months.
- The effects of twice daily consumption of green tea over 3 months was examined by one study [38].
- Daily consumption of 200 mg vitamin E over a period of 3 months [39]

All interventions were placebo controlled, excluding three [34,37,39]. Participant numbers varied from 37 subjects [28] to 360 [35]. The included participants were generally healthy patients with periodontitis. Factors like vitamin deficiencies or obesity were not in focus of the studies. The exact diagnosis was given in the minority of the studies; extraction of clinical data revealed that, in four interventions (vitamin E, supplementation of folic acid, oolong tea, and green Tea), predominantly patients with mild to moderate periodontitis were included [28,29,38]. Analysis of the data of all other studies revealed a treatment of patients with moderate to at least localized severe periodontitis.

### 3.3. Clinical Results of the Studies

#### 3.3.1. Pocket Probing Depths

Overall, eight studies reported a statistically significant reduction of mean probing depths: Babaei et al. [27] showed an improvement from 4.35 (SD = 0.08) mm to 2.12 (SD = 0.2) mm in the intervention group, compared to a significantly lower improvement from 4.48 (SD = 0.1) mm to 3.25 (SD = 0.06) mm in the control group. Keceli et al. [28] reported a statistically significant mean PPD reduction from 3.10 (SD = 0.56) to 2.26 (SD = 0.37) after 3 months in the folic acid group and from 3.12 (SD = 0.62) to 2.35 (SD = 0.60) in the placebo group. The adjunctive oolong tea intervention of Nafade et al. [29] revealed a drop of PPD from 6.13 (SD = 0.73) to 4.36 (SD = 0.85) in the test group and from 6.13 (SD = 0.89) to 4.63 (SD = 1.29) in the control group after 3 months. Both reductions were statistically significant. In Chapple et al. [30], the improvement was statistically significant only 2 months after intervention initiation for one of two test groups (fruit–vegetable group). After 5 and 8 months, the intergroup differences were no longer significant. In Chopra et al. [38], the green tea group showed a statistically significant reduction in mean PPD (*p* ˂ 0.001) and percentage of sites with PPD ≥ 4 mm compared to the control group after 1 and 3 months (mean reduction: test group 2.47 (SD = 0.64) mm, control group 1.91 (SD = 0.55 mm)). In the omega-3 intervention of Deore et al. [31], PPD reduction was significantly more pronounced after 12 weeks in the test group (4.26 (SD = 1.10) mm to 2.15 (SD = 0.53) mm) compared to the control group (4.05 (SD = 1.03) mm to 2.77 (SD = 0.47) mm). Investigating the adjunctive effect of a vitamin E supplementation, Singh et al. [39] found a significant PPD reduction in the test (median 3.73 (min. 2.38, max. 4.96 at baseline to median 1.85 (min. 1.14, max. 2.98) at 3 months follow-up) and control group (median 3.55 (min. 2.31, max. 4.33 at baseline to median 2.06 (min. 1.12, max. 3.03) at 3 months follow-up). The reduction was significantly more pronounced in the test group. In Gao et al. [35], vitamin D supplementation at 2000 IU and 1000 IU resulted in additional improvements in PD at probing depths ranging 4–6 mm compared to the control group, at 0.3 mm and 0.1 mm, respectively, after 3 months. At sites with PD >7 mm, groups receiving vitamin D showed additional reductions of 0.2 mm (2000 IU) and 0.1 mm (1000 IU) compared to the control group. Stańdo et al. [34] reported a mean PPD reduction of 1.3 (SD = 0.7) mm for the intervention group (20 mL of fish oil per day) which was comparable to the control group (1.1 (SD = 0.4) mm). Additionally, they analyzed the percentage of closed pockets, defined as PPD ≤ 4 mm without BOP and reported a statistically significant difference to the control group (intervention: 58 (SD = 17)% compared to control 49 (SD = 11)%). Regarding the reduction of pathological pockets (PPD ≥ 4 mm), there were no differences between the groups.

#### 3.3.2. Bleeding on Probing

The authors of six studies reported a statistically significant improvement in percentage of sites with bleeding on probing (BOP) in the test group compared to the control group [26,28,30,37,38,39]. The most pronounced results were reported by Singh et al. [39]. BOP dropped in the Test group around 60% from median 67.35% (min. 51.46%, max. 76.39%) to 6.18% (1.28%, 12.69%). This was significantly higher compared to the control group with a BOP reduction of 47.80% (38.12%, 58.67%). Chopra et al. [38] found a near 20% greater reduction compared to the control group (test group: 93.31 (SD = 5.79)% to 21.0 (SD = 6.35)%; control group: 88.84 (SD = 5.76)% to 31.2 (SD = 8.88)%) after 3 months. In Arora et al. [26], after an observation period of 2 months, the lycopene intervention resulted in a significant decrease in BOP compared to the control group (1.12 (SD = 0.138)% vs. 1.02 (SD = 0.144)%, respectively). Intergroup comparison to the placebo group showed a statistically significant reduction in BOP in the lycopene group. Nafade et al. [29] reported a significant BOP reduction in the test group from 83.41 (SD = 21.08)% to 32.86 (SD = 11.12)% after 3 months. The reduction was also significant in the non-oolong tea control group with no significant intergroup differences. In Chapple et al. [30], BOP decreased in all groups. A significantly lower value for the fruit–vegetable group was only observed at month 5. In Graziani et al. [37], after 2 months of diet-only intervention, the Full Mouth Bleeding score was significantly decreased compared to the control group (55.30 (SD = 17.82) vs. 48.64 (SD = 18.64), respectively). After additional subgingival instrumentation, both interventions improved their scores compared to baseline. However, at the Month 5 endpoint, the test group showed significantly higher BOP values. Within the study of Stańdo et al. [34], the BOP was significantly lower after 3 months compared to the control (14 (SD = 6)% vs. 21 (SD = 7)%, respectively). The reduction did not differ between the groups.

#### 3.3.3. Clinical Attachment Loss

Six studies reported a statistically significant CAL reduction within the test groups compared to the control groups. In the study of Keceli et al. [28], the CAL dropped from 3.35 (SD = 0.67) to 2.64 (SD = 0.37). It dropped significantly in the placebo group as well: 3.53 (SD = 0.76) to 2.96 (SD = 0.63). Chopra et al. [38] reported an average attachment gain of 2.47 (SD = 0.64) mm in the green tea group from baseline to month 3. After 12 weeks, Deore et al. [31] found a significantly lower CAL in the omega-3 group (5.53 (SD = 0.95) mm to 2.73 (SD = 0.98) mm). Likewise, after 12 weeks, Singh et al. [39] found a significant lower CAL in both groups (Test group: median 4.12, min. 1.66, max. 6.50 at Baseline to median 2.34 (min. 0.95, max. 6.20) at Follow-up; Control group: 3.82 (1.91, 6.79) at Baseline to 2.85 (0.57, 5.59) at 3 months follow-up) with a significantly greater reduction in the test group. Gao et al. [35] reported a 0.1 mm greater attachment gain within the intervention groups (vitamin D3 2000 IU and 1000 IU) compared to the control. In concordance with the BOP results, Graziani et al. [37] reported a statistically significant CAL reduction within the kiwifruit test group at the end of the diet-only period. After subgingival instrumentation, the control group showed a statistically lower CAL. Three months after SRP, the CAL gain was significantly more pronounced within the fish oil group (1.3 (SD = 0.7) mm) of Stańdo et al. [34] compared to the control (0.9 (SD = 0.4) mm).

#### 3.3.4. Total Antioxidant Capacity

Two studies reported a statistically significant increase in total antioxidant capacity (TAC), defined as the capacity of antioxidants to act against oxidative stress and one study measured the total antioxidants in serum, saliva, and GCF [40]. Babaei et al. [27] measured serum TAC using a commercial kit and reported an increase within the test group (pre: 1.50 (SD = 0.35), post: 1.89 (SD = 0.49); *p* ˂ 0.001) after 2 months, compared to a decrease in the control group. Similarly, Chopra et al. [38] showed an 8.02 (SD = 4.76)-fold increase in antioxidant capacity in gingival crevicular fluid compared to the control group, and a 12.06% increase relative to baseline in the blood, compared to a decrease of 2.76% in the control group using a Ferric-Reducing Antioxidant Power Assay (FRAP) to analyze GCF- and plasma TAC. The duration of the intervention was 3 months. Nafade et al. [29] measured the total antioxidants in serum, saliva, and GCF, measuring its ferric-reducing antioxidant power, and found a significant increase in the oolong tea group and in the non-oolong tea group as well.

#### 3.3.5. Cytokines in Serum and Saliva

##### TNF-α

Two studies analyzed salivary TNF-α levels. Keskiner et al. [32] showed a significant reduction after 6 months in both the test (from median 38.18 (27.89–44.24) pg/mL to 11.86 (9.74–16.75) pg/mL) and control group (Baseline 32.43 (27.50–43.05) pg/mL to 20.59 (18.19–22.89) pg/mL). The results were significantly significant for the test group. In contrast, Arora et al. [26] did not find a significant improvement in serum TNF-α. 

##### CRP

Keceli et al. [28], Deore et al. [31], and Martinez et al. [33] reported serum levels of *C*-reactive protein (CRP) and found no significant differences. 

##### Interleukins

Stańdo et al. [34] analyzed the evolution of a number of pro- and anti-inflammatory cytokines and found a significant reduction of proinflammatory IL-12 and IL-17 compared to the control. The anti-inflammatory cytokine IL-10 increased significantly in intergroup comparison. 

##### Chemokines

Regarding chemokines, Stando et al. [34] reported that CCL-5/RANTES, CCL22/MDC, CCL25TECK, CX3CL1/Factalkine, and CXCL8/IL-8 increased compared to the control.

#### 3.3.6. Serum Lipid Levels

The chicory intervention [27] resulted in a significant decrease in low-density lipoprotein (LDL) levels from 207.51 (SD = 67.92) to 155.65 (SD = 61.81) mg/dL, and an increase in high-density lipoprotein (HDL) levels from 32.15 (SD = 6.41) to 42.25 (SD = 8.47) mg/dL after 2 months. No significant differences were reported in the control group. In contrast, within a timeframe of 5 months, the kiwifruit intervention of Graziani et al. [37] found a significant decrease in HDL levels (3.33 (SD = 14.51) mmol/L) compared to the control group. In addition, HDL levels increased by 4.37 (SD = 7.41) mmol/L in the control group. Martinez et al. [33] and Perić et al. [36] also analyzed HDL levels, but did not find significant differences between the groups. The characteristics and results of the included studies are summarized in Table 1.

#### 3.3.7. Superoxide Dismutase (SOD) Activity in Serum and Saliva

Singh et al. [39] investigated the activity of SOD, representing an important antioxidant enzyme. It was measured in saliva and serum before and after treatment. Saliva and serum SOD activity increased in both groups significantly groups (Saliva: Test group, median 11.19%, min. 1.75, max. 55.68 at Baseline to median 14.36 (min. 3.53, max. 57.93) at Follow-up; Control group: 10.71 (0.73, 42.15) at Baseline to 17.27 (6.67, 55.23) at 3 months follow-up; Serum: Test group, median 61.06%, min. 10.31, max. 85.18 at Baseline to median 85.90 (min. 49.06, max. 57.39) at Follow-up; Control group: 59.41 (23.43, 85.65) at Baseline to 62.06 (55.19, 90.92) at 3 months follow-up). Intergroup comparison revealed no differences between the groups for saliva SOD activity and a significant, more pronounced increase of serum SOD activity in the Test group compared to the Control group.

### 3.4. Risk of Bias

**Figure 2:**
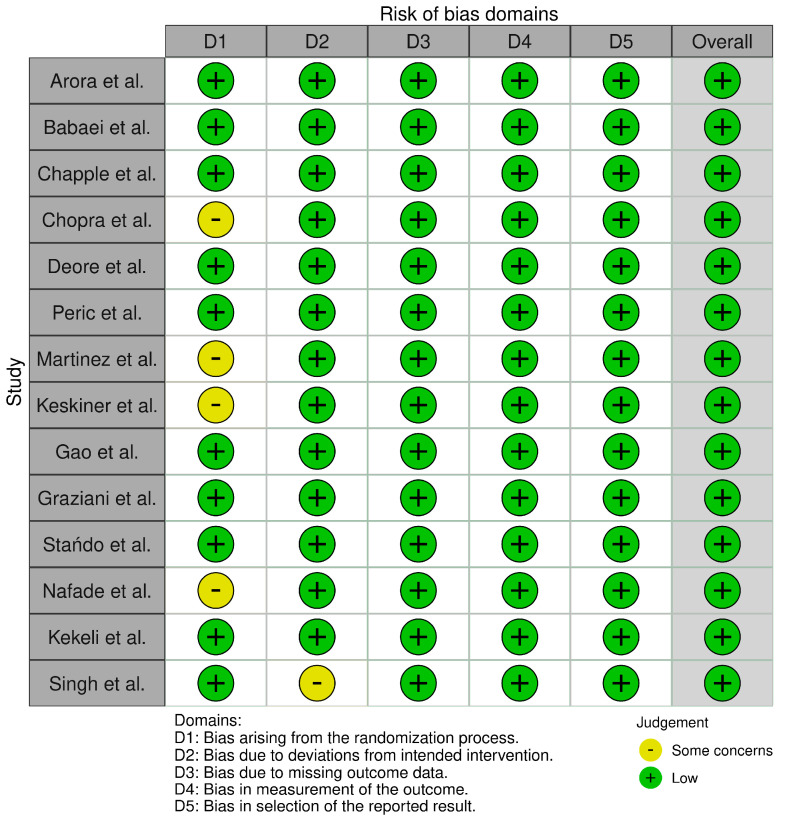
Risk of Bias analysis according to RoB 2: Domains.

**Figure 3:**
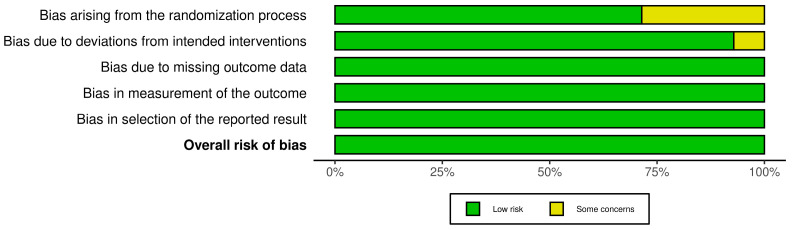
Risk of bias assessment according to RoB2: Overall rating.

The risk of bias analysis according to RoB 2 is shown in Figure 2 and Figure 3. Most of the studies showed a low risk of bias. Some concerns were found for imprecise reporting on the randomization process or registration of the study in an international trials registry.

### 3.5. Meta-Analysis and Grading of Evidence

Due to a limited data availability, regarding the within-groups differences, no useful meta-analysis or grading (GRADE) was possible. Of the 14 included studies, only 5 studies reported within-group differences on some clinical parameters on different nutraceuticals [26,34,37,38,39]. All corresponding authors from studies investigating the same nutraceutical (omega-3 fatty acids, vitamin D, green/oolong tea) were contacted via email. Only one corresponding author answered, but they were not able to deliver the needed within-group differences. 

However, a preliminary meta-analysis was performed for all investigated nutraceutical approaches with a data report for the differences between baseline and 3 months follow-up. In total, four studies could be included for PPD and CAL [26,34,37,38]. Due to a lack of BOP data, the study of Chopra et al. [38] could not be included into the meta-analysis for BOP. Moreover, Singh et al. [39] reported the median values with minimum and maximum. Hence, the calculation included the outcomes for the adjunctive use of omega-3 fatty acids, green tea, kiwifruit, and lycopene. Overall, all investigated outcomes were in favor for the nutraceutical approaches (*p* < 0.001) but showed a high heterogeneity, especially for PPD and BOP. The results of the preliminary meta-analysis are provided in Supplementary Table S2 and Figure S1.

Accordingly, an attempt was also made to grade the quality of evidence assessment by using GRADE [41]. Due to the limited data and no sufficient meta-analysis, this was not fully possible (with regard to the missing summarized confidence interval). However, a preliminary approach showed very low evidence for most of the neutraceutical outcomes. Due to the number of participants with regard to the topic of vitamin D, omega-3 fatty acids, and green/oolong tea, these topics were rated with low evidence (Table 2). With regard to the field of nutraceuticals, there was a minor indirectness in most of the studies based on the fact that these interventions might only work in patients how have deficiencies or low levels of this nutrient.

## 4. Discussion

This systematic review investigated the effects of nutritional interventions in combination with subgingival instrumentation in patients with periodontitis. Ten out of fourteen included RCTs demonstrated positive effects on periodontal parameters, including PPD, BOP, and CAL. These studies involved interventions with lycopene, chicory extract, fruit–vegetable–berry capsules, green tea, oolong tea, folic acid, vitamin D3, vitamin E, and omega-3 fatty acids. Overall, 3 of 14 studies did not show statistically significant benefits of nutritional interventions, including vitamin D3 and omega-3 fatty acids. An intervention with kiwifruits reported a better outcome for PPD in the control group after subgingival instrumentation; however, in terms of BOP, the authors reported a statistically significant therapeutic effect of the kiwifruit consumption.

All studies included healthy participants with periodontitis; only one study [36] reported a baseline maximum value for vitamin D within the inclusion criteria. However, this was not in the range of a manifest deficiency. This is important to mention because, in general, nutraceutical approaches provide defined nutrients and it can be assumed that a therapeutic effect would be more pronounced in cases with deficiencies or low levels of the provided nutrients.

### 4.1. Nutraceutical Interventions and Their Mechanisms in Context to the Literature

The main effect of antioxidants is to decrease oxidative stress. Numerous studies have reported the presence of increased oxidative stress markers and decreased antioxidant levels in the serum or saliva of patients with periodontitis [42,43,44]. Increased oxidative stress and the activation of nucleus factor kappa b (NF-κB) pathways is associated with the upregulated expression of genes related to pro-inflammatory cytokines [45]. It can be assumed that all included studies had antioxidant effects. Three studies [27,29,38] recorded TAC, TAO, or SOD in serum and saliva, respectively, with all showing significantly increases. This underlines recommendations of recent reviews regarding supportive treatment of periodontitis with antioxidant administration [43,46].

The described amounts of polyphenols and flavonoids in the two tea studies [29,38] are comparable to other works [47,48] and in line with results of a study which reported that flavonoid intake through fruits and vegetables was associated with lower saliva IL-1β and reduced PPD during supportive periodontal care [49].

The daily prescribed dose of the fruit–vegetable capsules in the study of Chapple et al. [30] contained 7.5 mg β-carotene, 46 mg vitamin E, 200 mg vitamin C, and 400 µg folic acid. Compared with placebo, the capsules significantly reduced PPD and BOP after 2 and 5 months, respectively. Studies using the same compounds reported positive effects on markers for oxidative stress and pro-inflammatory cytokines such as TNF-α [50,51]. The fruit–vegetable–berry group of the same study consumed even higher doses of vitamin C, E, and folic acid (66 mg vitamin E, 222 mg vitamin C, and 640 µg folic acid). However, this did not further affect periodontal indices [30]. A polymorphism in the gene encoding β-carotene monooxygenase, which may be partly responsible for the low bioavailability of the active form of β-carotene, was discussed as potential reason for the minimal effects observed in this intervention group. This mechanism has been previously described by [52]. According to Chapple et al. [30], poor absorption via the gastrointestinal tract and interfering ingredients could also further explain these results. 

Graziani et al. [37] demonstrated a statistically significant improvement in PPD reduction after two months of kiwifruit consumption and without subgingival instrumentation, but not after additional SRP. The authors discussed a potential masking of the kiwifruit consumption effects by the strong effect of subgingival instrumentation [37]. Accordingly, kiwifruits might have a certain positive effect against periodontal inflammation, which is, however, much less than the strong effect of subgingival inflammation. Kiwifruits are a source for vitamin C, which is known to serve at an antioxidant capacity [53]. Legott et al. [54] observed the alternate consumption of a vitamin-C-rich or -poor diet over a period of 3 months. Although serum vitamin C levels reflected the alternating diet, increased administration of vitamin C improved gingival inflammation. This is in contrast to the results of Woelber et al. [18], demonstrating an effect of a diet rich in antioxidants on BOP, but not on gingival index. Amaliya et al. reported an effect of 200 mg vitamin C during a 14-day period in the absence of oral hygiene. Vitamin C intake either through guava fruit or synthetic vitamin C led to a lower increase of GI compared to the control group [55]. Interestingly there is no study investigating the additional effect of vitamin C to non-surgical periodontal therapy.

Another antioxidant intervention was conducted by Singh et al. [39]. They gave 200 mg vitamin E over a period of three months with significant beneficial effects on almost all periodontal parameters compared to the control group. The role of vitamin E as an antioxidant and its role in regulation of immunological responses has been widely studied [56,57,58]. However, only a small number of studies investigated the effect of Vitamin E on periodontitis so far [59]. Proposed mechanisms are the mentioned antioxidant effects on ROS, and, additionally, it seems to reduce the levels of proinflammatory cytokines and inhibit the expression of COX-2 [59].

Vitamin D3 was used in the studies by Gao et al. [35] and Perić et al. [36] with different dosages and time periods. Gao et al. [35] investigated two different intervention groups, taking either 2000 IU or 1000 IU of vitamin D3 per day. Periodontal examination parameters were moderately improved, with significant differences between the intervention and control groups. In contrast, Perić et al. [36] gave an ampoule of 25,000 IU vitamin D3 per week, showing no significant differences with periodontal parameters after 6 months. The doses administered in Gao et al. [35] were equivalent to 14,000 and 7000 IU per week, respectively, which was lower than the doses given by Perić et al. [36]. However, the results suggest that the lower doses were more effective. One explanation for these results could be that Gao et al. [35] investigated a study cohort of 360 subjects, compared to the low number of 27 participants in Perić et al. [36]. Whether vitamin D is given daily or weekly did not affect the outcome of previous studies [60,61,62]. A systematic review by Machado et al. [63] showed that vitamin D3 levels were significantly lower in patients with periodontitis compared to healthy controls.

One study investigated the adjunctive effects of folic acid capsules adjunctive to periodontal therapy [28] with a significant higher reduction of BOP within the test group. Deficiencies of folic acid have been shown to be linked to the severity of periodontal disease [64,65,66]. The mechanism is not well understood so far; however, beneath other negative effects, folic acid deficiencies have been reported as related to oxidative stress, a major driver of inflammatory processes [67]. Due to a lack of intervention studies with folic acid in patients with periodontitis, the used dosage cannot be compared to dosages in other periodontal studies with the use of folic acid. Furthermore, the included study population was not suffering from folate deficiencies. 

Four studies investigated the anti-inflammatory effects of omega-3 fatty acids, which are precursors of several anti-inflammatory factors, such as anti-inflammatory cytokines or resolvins. On the other hand, omega-6 fatty acids are precursors of multiple pro-inflammatory factors; hence, an omega-6/omega-3 intake ratio of 4:1 to 1:1 is recommended [68]. The differences in the results between the included omega-3 studies could be explained by highly varying amounts of omega-3 from around 25 mg in Keskiner et al. [32] to 4400 mg in Stańdo et al. [34]. The effects of omega-3 fatty acids on periodontitis have been examined in various studies. In an epidemiological study with over 9000 participants, an inverse correlation between omega-3 intake and the incidence of periodontitis was reported [69]. Three systematic reviews on the adjunctive use of Omega-3 to non-surgical periodontal therapy concluded that omega-3 fatty acids had a statistically significant effect on reduction of PPD and CAL when given in addition to subgingival instrumentation [70,71,72]. Based on the results of their study, van Ravensteijn et al. recommended a minimum of 2 g omega-3 per day when used adjunctive to non-surgical periodontal therapy [71]. However, some of the included studies prescribed low-dose aspirin as a further pharmacological intervention. The studies with the additional use of aspirin were excluded in the current methodology because this intervention cannot be considered as a nutraceutical anymore. In addition to the missing within-group differences of the other studies, only one study remained to be able for a further meta-analysis. Considering that the omega-fatty acids ratio plays an important role, background nutrition should be adjusted and monitored when omega-3 fatty acids are given as a supplement. Within a Western diet including a high intake of meat and arachidonic acid, it may be difficult to achieve an omega-6 to omega-3 ratio of 4:1 or lower. Hence, without further data on the diet of participants, the validity of the results may be questioned.

### 4.2. Biochemical Parameters

In addition to clinical periodontal parameters, two studies investigated TAC and SOD [27,38] and reported beneficial effects, in line with previous conducted studies on green tea and chicory [73,74,75]. Despite the fact that numerous studies have shown an increase in CRP levels in patients with periodontitis [76,77], neither of the included studies showed significant effects on CRP. Regarding other pro-inflammatory cytokines, the omega-3 study of Keskiner et al. [32] reported a reduction of TNF-α levels in the intervention group that was not observed in the control group. Regarding TNF-α levels, a recent meta-analysis was not able to show a significant reduction after subgingival instrumentation [78]. Decreased serum HDL levels have been reported in patients with periodontitis [79]. Moreover, HDL values may improve after subgingival instrumentation [80]. Only the chicory study reported a significant increase in HDL levels after intervention compared to the control group [27]. No significant differences were found between the test and control groups in two additional studies investigating HDL levels after intervention with omega-3 and vitamin D3 [33,36]. Within the kiwifruit study, a negative trend was reported within the test group, but this was not significant [37]. 

In general, absorption capacity and bioavailability can be influenced by factors like medication, gene polymorphisms, and interaction between different nutrients [81]. It remains unclear whether those interactions may have influenced the intervention effect in studies utilizing micronutrient containing capsules [26,30,82,83]. Moreover, the effects of supplemental nutrients might be overlaid by dietary behaviors, such as Western diet patterns [84,85].

### 4.3. Considerations Regarding the Use of Nutraceuticals

It must be mentioned that, in contrast to medication, none of the included studies reported any adverse effects. Eight studies demonstrated a significant effect on periodontal inflammatory parameters, systemic inflammation, and lipid levels. Nevertheless, the use of encapsulated nutrient supplements encompasses the danger of micronutrient overdosing. On the other hand, micronutrient capsules, in general, may only claim to deliver an adequate amount of micronutrients and concurrently lower the awareness for micronutrient intake through food. Comparing the micronutrient content of the products and foods investigated within the presented RCTs with natural foods containing comparable nutrient amounts illustrates a large degree of heterogeneity. This underlines the importance of a micronutrient-rich base diet (Supplementary Table S2). Taken together with the above discussed factors of low-quality background diet and potential nutrient interactions, a generally healthy diet with natural foods may provide many advantages compared with the use of supplements.

### 4.4. Limitations

Regarding background nutrition assessment, only Martinez et al. [33] used a questionnaire. Assessing background diet is also important for all other dental research looking at clinical parameters, as diet might cause effect size biases [86]. Despite the fact that we exclusively included RCTs and excluded systemically predisposed patients as well as patients with regular medications, the small numbers of participants and short observation periods may be a limitation in general. In addition, the heterogeneity of these nutritional interventions, which included different foods, supplements, and dosages, as well as different severities and definitions of periodontitis, complicated a comparison of the results. Hence, these points may be important considerations for the planning of future studies: precise dosage information, duration of the intervention, documentation of the background diet, bioavailability and possible interactions of nutrients, and a possible bias effect caused by additional treatment. Furthermore, with regard to the chosen inclusion and exclusion criteria, it can be discussed if the use of nutraceuticals should be limited to non-locally applied substances. This strategy was chosen in order to present more useful evidence for daily application in the life of periodontitis patients. Nonetheless, supplements such as vitamin B12 are already offered as ingredients in dentifrices. 

Furthermore, there was a surprising insufficient reporting of within-group differences in most of the studies (10 of 14 studies), which makes it challenging to use these data for a further meta-analysis. Regarding methodological factors in nutritional studies, it has to be mentioned that a whole diet change or the intake of certain foods like kiwifruits cannot be blinded for the participants, especially if the control group is asked to keep their habitual diet. Moreover, randomization procedures could be discussed in this context. Participants with motivation to change their diet might be disappointed to be allocated to a control group, possibly affecting subconsciously their behavior. In the case of studies investigating the effect of a nutritional intervention as an adjunct to non-surgical periodontal therapy, it might be difficult to determine the effect that is caused by the nutritional intervention alone; mechanical periodontal therapy might mask the effect of the nutritional intervention in an unknown dimension, as shown in Graziani et al. [37]. However, if the topic of the study is the adjunctive effect of the nutritional intervention, this might not be the primary interest. Another limitation of the present study is that the search term formulation could have been more precise, for example, including antioxidants and other substances. However, a more specific search term might be difficult because every single micronutrient, polyphenol, or phytochemical would be necessary. Another systematic review on the same topic included only four articles [87] with two non-randomized trials and a study by Javid et al. [88], which lacked complete data on the periodontal examination parameters. For this reason, we excluded this study. 

We conducted a preliminary meta-analysis to get a theoretical view into the ’black box’ of potential effects of nutraceutical intervention in total. However, the study focuses on the adjunctive effect of nutraceutical and dietary interventions, and such a preliminary analysis could provide a general direction on how to estimate the effect of such an approach. From a practical point of view, it accordingly might be beneficial to combine interventions with different dietary and nutraceutical interventions. Regarding literature search methodology, a strength of our study was the use of PICO, the GRADE tool to assess evidence, and the Cochrane Collaboration’s tool for assessing risk of bias. In the present work, only RCTs were included.

To assess the level of quality of the non-surgical periodontal treatment, we compared the results of the control treatments with those of the meta-analysis in the EFP-S3 guideline [6], indicating comparable PPD reductions. Hence, the results of the control treatments seem to be in line with the results of standard therapy outcomes.

In conclusion, there is some evidence for the effect of adjunctive nutritional interventions in patients undergoing non-surgical periodontal therapy. We found a high heterogeneity in the type of intervention and all eleven studies used supplementary or nutraceutical approaches. However, the majority of the studies reported a positive effect of the different nutritional interventions on clinical periodontal and systemic inflammation. The adjunctive use of various supplements and green/oolong tea led to positive and significant effects of the nutritional interventions on clinical periodontal outcomes and systemic TNF-alpha levels and antioxidant capacity. In the context of non-surgical periodontal therapy, an adjunctive supplementation with micronutrients (at least 300 mg of omega-3 fatty acids), green/oolong tea, and antioxidants could be beneficial. Long-term clinical studies with larger numbers of subjects, a homogenous definition of periodontitis and its severity, and a background diet assessment in case of diet supplements, as well as a full report of frequencies (number of closed pockets) and within-group differences are needed to confirm these findings and to allow a further meta-analysis.

## Figures and Tables

**Table 1:** Characteristics of the included studies.

**First Author**	**Arora et al.**	**Babaei et al.**	**Keceli et al.**	**Nafade et al.**	**Chapple et al.**	**Chopra et al.**	**Graziani et al.**
**Publication year**	2013	2018	2019	2022	2012	2016	2017
***n* total** ***n* control** ***n* intervention**	46 23 23	40 20 20	37 20 17	60 30 30	61 21 Je 20	120 60 60	50 25 25
**Study design**	RCT	RCT	RCT	RCT	RCT	RCT	RCT
**Intervention group**	8 mg lycopene daily (2× daily 2 softgel capsules with 2 mg each) in the form of lycopene vitamin mineral capsules + SRP	2× daily capsules with 1 g chicory extract + twice SRP	400 mcg folic acid with calcium and vitamin D, 1× daily + single SRP	4 g oolong tea per day for 30 days + SRP	2× daily 3 fruit-vegetable or fruit-vegetable-berry juice powder capsules for 9 months + SRP	2× daily a cup of green tea for + SRP	2× daily one kiwi + SRP after 2 months
**Control group**	2× daily 2 placebo each + SRP	2× daily placebo for 8 weeks + twice SRP	1× daily placebo + single SRP	No oolong tea + SRP	2× daily 3 placebo + SRP	2× daily a cup of cellulose tea (placebo) + SRP	No kiwis + SRP
**Type of intervention**	Lycopene vitamin mineral capsules + SRP	chicory extract capsules + twice SRP	Folic acid capsules + SRP	Oolong tea + SRP	Fruit-vegetable or fruit-vegetable-berry juice powder capsules + SRP	green tea + SRP	Kiwifruit + SRP
**Duration of intervention**	2 months	2 months	6 months	3 months	9 months	3 months	5 months
**Who carried out intervention?**	Periodontist	Dentist	n.a.	n.a.	n.a.	n.a.	n.a.
**Examination interval**	Baseline, every 15 days until endpoint at 2 months.	Baseline, after 8 weeks	Baseline, after 1, 3, and 6 months	Baseline, after 1, 3 months	Baseline, after 2, 5, and 8 months.	Baseline, after 1 and 3 months	Baseline, 2, 3, 4, and 5 months.
**Outcomes**	PPD, CAL, salivary IL1ß, BOP, PI, MGI, serum TNFα, salivary uric acid.	PPD, BMI, TAC, MDA, uric acid, blood lipids (TC, TG, LDL-C, HDL-C)	PPD, CAL, PI, GI, GR, serum folic acid, GCF volume, Homocysteine, CRP	PPD, CAL, BOP, GI, PI, total antioxidants, glutathione peroxidase, malondialdehyde, CFUs in Plaque	PPD, CAL, BOP, GCF, gingival redness: MGI, cumulative plaque index (Lobene et al. 1982), recessions.	PPD, CAL, BI, GI, PI, antioxidant levels in GCF and plasma.	PPD, CAL, BOP, FMPS recessions, BMI, serum markers: CRP, HbA1c, HDL, LDL, TG, TC, serum vitamin C.
**Drop-outs**	Yes (*n* = 4)	No	Yes (*n* = 23)	n.a.	Yes (*n* = 6)	Yes (*n* = 5)	No
**Results compared with test group**	↓PI, ↓MGI, ↓BOP ↓salivary IL1ß, ↓uric acid levels.	↓PPD, ↑TAC	After 3 months: no sig. diff for PPD, GR; GI and PI, GCF and CRP, ↓CAL, ↑folic acid, ↓Homocysteine	After 3 months: no sig. diff for PPD and CAL, ↓PI, ↓GI, ↓BOP, ↑total antioxidants, ↓glutathione ↓peroxidase, ↓malondialdehyde, ↓CFUs in Plaque	After 2 months: ↓PPD (FV) After 5 months: ↓BOP (FV) ↓GCF (FVB).	↓ PPD, ↓BOP, ↑TAC in sulcus fluid.	↓CAL, FMPS, and FMBS of control group ↓Blood pressure, ↑Vitamin C.
**Conclusions**	+	+	+	+	+	+	-
**First Author**	**Deore et al.**	**Stando et al.**	**Keskiner et al.**	**Martinez et al.**	**Singh et al.**	**Gao et al.**	**Peric et al.**
**Publication year**	2014	2020	2017	2014	2014	2020	2020
***n* total** ***n* control** ***n* intervention**	60 30 30	40 20 20	30 15 15	15 8 7	38 19 19	360 120 240	27 14 13
**Study design**	RCT	RCT	RCT	RCT	RCT	RCT	RCT
**Intervention group**	1× daily 300 mg omega-3 fatty acids 180 mg EPA and 120 mg DHA + SRP	2× daily 10 mL of fish oil (=2.6 g EPA, 1.8 g DHA, 1.4 g alkylglycerol, 1.4 g squalene, 240 µg vitamin A, 2 µg vitamin D3) + SRP	2× daily low dose Omega 3 PUFA (6.25 mg EPA and 19.19 mg DHA in the form of fish oil) + SRP	3× daily 300 mg omega-3 FS capsules (180 mg EPA, 120 mg DHA) for 12 months) + SRP	1× daily 200 mg vitamin E + SRP	1× daily 2000 IU resp. 1000 IU vitamin D3 + SRP	1× weekly ampoule 25,000 IU vitamin D3 (=approx. 3571 IU daily) + SRP
**Control group**	1× daily placebo: 300 mg liquid paraffin + SRP	SRP	2× daily placebo + SRP	3× daily placebo + SRP	SRP	1× daily placebo + SRP	1× weekly placebo + SRP
**Type of intervention**	Omega-3 fatty acids + SRP	Fish oil + SRP	Omega 3 fatty acids + SRP	Omega-3 fatty acids + SRP	Vitamin E + SRP	Vitamin D3 + SRP	Vitamin D3 + SRP
**Duration of intervention**	3 months	3 months	6 months	12 months	3 months	3 months	6 months
**Who carried out intervention?**	Dentist/periodontist	Dentist/periodontist	n.a.	Nutritionist and periodontist	n.a.	n.a.	n.a.
**Examination interval**	Baseline, 6 and 12 weeks	Baseline, after 3 months	Baseline, 1, 3, and 6 months	Baseline 4 and 12 months	Baseline, after 3 months	Baseline, after 3 months	Baseline, after 1, 3, and 6 months
**Outcomes**	PPD, CAL, SBI, PI, GI, OHIS (Oral Hygiene Index Simplified), serum CRP	*n* PPD ≥ 4 mm, PPD, CAL, REC, BOP, % of PPD ≤ 4 mm without BOP, Salivary cytokines, chemokines and growth factors	PPD; CAL BOP, PI, GI, saliva: TNF-α, SOD.	PPD, CAL, BOP, BMI, blood: lipid levels, CRP, omega-3 and omega-6 LC-PUFA (EPA, DHA, DPA, AA), plasma glucose, TAC, TC, HDL-c, LDL-c, VLDL-C, HbA-1c, insulin, and leukocytes.	PPD, CAL, BOP, GI, PI, Saliva SOD activity, Serum SOD activity	PPD, CAL, BI, PI, serum 25(OH)D level, ACH (alveolar crest height)	PPD, FMBS, FMPS, creatinine, osteocalcin, alkaline phosphatase, calcium, HDL, parathyroid hormone, neutrophils, lymphocytes
**Drop-outs**	Yes (*n* = 2)	Yes (*n* = 10)	No	No	No	Yes (*n* = 37)	Yes (*n* = 6)
**Results compared with test group**	↓GI, ↓SBI, ↓PPD ↓CAL serum CRP no sig. Change	↑REC, ↓CAL, ↓BOP, ↓FMPI ↑∆CAL, ↑∆% of PPD ≤ 4 mm without BOP, ↓IL-12, ↓IL-17, ↑IL-10, ↑CCL-5/RANTES, ↑CCL22/MDC, ↑CCL25TECK, ↑CX3CL1/Factalkine ↑CXCL8/IL-8 ↑FGF2, G-CSF	Perio parameters no sig. Differences between groups↓, TNF-α.	Perio parameters no sig. Differences, ↑EPA, ↓BOP in control group.	↓PPD, ↓CAL, ↓BOP, ↓GI, ↓PI, ↓Saliva SOD activity, ↓Serum SOD activity in both groups ↑∆PPD, ↑∆CAL, ↑∆BOP, ↑∆GI, ↑∆PI, ↑∆Saliva ↑∆SOD, ↑∆Serum SOD	↓AL, ↓PPD	Perio parameters no sig. Differences
**Conclusions**	+	+	-	-	+	+	-

Note PPD = pocket probing depth, CAL = clinical attachment loss, BOP = bleeding on probing, GI = gingival index, FMBS = full mouth bleeding score, FMPS = full mouth plaque score, TAC = total antioxidant capacity, CRP = C-reactive protein, SRP = scaling and root planing, EPA = eicosapentaenoic acid, DHA = docosahexaenoic acid, SOD = superoxide dismutase, RCT = randomized controlled trial, ↑/↓ = statistically significant decrease, resp. increase in comparison of test/control group.

**Table 2:** Grading of Recommendations Assessment, Development and Evaluation (GRADE) according to Schünemann 2009.

Quality Assessment								Summary of Findings			
								*N* Patients		Effect	Evidence
Topic	*N* Studies	Design (RCT)	Limitations/Risk of Bias	Inconsistency	Indirectness	Imprecision	Other Considerations	Experimental Group	Control Group	SMD [95% Conf. Interval]	
Vitamin D	2	Yes	No serious limitations	Some inconsistency	Some risk of indirectness * 1	n/a		225	119	n/a	Low
Omega-3 fatty acids	4	Yes	No serious limitations	Some inconsistency	Some risk of indirectness * 1	n/a		67	66	n/a	Low
Kiwi-fruit	1	Yes	No serious limitations	No serious inconsistency	Some risk of indirectness * 1	No serious risk of imprecision		25	25	PPD: −1.62 [−2.27–−0.99]	Very low
Grean tea/Oolong tea	2	Yes	Some limitations * 2	No serious inconsistency	Some risk of indirectness * 1	n/a	PROMs: less need for reinstrumentation	86	89	n/a	Low
Fruit & vegetable capsules	1	Yes	No serious limitations	Some inconsistency * 3	Some risk of indirectness * 1	n/a		20	20	n/a	Very low
Chicory extract	1	Yes	No serious limitations	No serious inconsistency	No serious indirectness	n/a		20	20	n/a	Very low
Lycopene mineral caspules	1	Yes	No serious limitations	No serious inconsistency	Some risk of indirectness * 1	No serious risk of imprecision		21	21	PPD: 0.00 [−0.61–0.61]	Very low
Folate	1	Yes	Some limitations * 4	No serious inconsistency	Some risk of indirectness * 1	n/a		20	17		Very low
Vitamin E	1	Yes	No serious limitations	No serious inconsistency	Some risk of indirectness * 1	n/a		19	19		Very low

* 1 May only work in nutrient/antioxidant-deficient patients. * 2 Due to limited randomization, not applicable blinding, only mild to moderate periodontitis. * 3 Different outcomes between fruit/vegetables and fruit/vegetables/berry capsules. * 4 High drop-out rate.

## Data Availability

The data that support the findings of this study are available from the corresponding author upon reasonable request.

## Supplementary Materials

The following supporting information can be downloaded at: https://www.mdpi.com/article/10.3390/nu15061538/s1, Table S1: Excluded studies according to full text analysis; Table S2: Data of the meta-analysis including all studies providing values of difference between baseline and three months follow up: (a) PPD, (b) CAL, (c) BOP; Table S3: Comparison of supplemented micronutrients and food intake possibilities; Figure S1: Forest plots for the outcomes of (a) PPD, (b) CAL and (c) BOP.
