# Supplementary material for: Dietary and Nutraceutical Interventions as an Adjunct to Non-Surgical Periodontal Therapy—A Systematic Review
Source: Nutrients. 2023 Mar 22;15(6):1538. doi: 10.3390/nu15061538 (PMC10052653; doi:10.3390/nu15061538)
Supplement: Supplementary file 1 [file nutrients-15-01538-s001.zip › nutrients-2268565-supplementary.pdf]

Supplementary

Supplementary Table S1: Excluded studies according to full text analysis.

Author, year	Literature search	Reason for exclusion
Kondo et al. (2014)	E	N2
Fridell et al. (2017)	E	N1
El-Shinnawi et al. (2015)	E	N3
Woelber et al. (2016)	E	N1
Uhrbom et al. (1984)	E	N1
Cocate et al. (2019)	E	N2
Al-Ahmad et al. (2010)	E	N1
Melcher et al. (1953)	E	N3
Wozniewicz et al. (2018)	E	N5
Isola et al. (2020)	E	N3
Fourel et al. (1972)	E	N3
Silvestrini et al. (1954)	E	N3
Dawson et al. (2014)	E	N3
Lau et al. (2013)	E	N3
Martinez-Herrera (et al. (2018)	E	N2
Mamae-Chomata et al. (1979)	E	N3
Leggott et al. (1991)	E	N1
Maruhashi et al. (1983)	E	N3
Rasperini et al. (2019)	E	N1
Harpenau et al. (2011)	E	N1
Neiva et al. (2003)	E	N3
Leger et al. (1965)	E	N3
Bradford et al. (1959)	E	N3
Cheraskin et al. (1988)	E	N1
Zare Javid et al. (2018)	E	N2
Zare Javid et al. (2019)	E	N2
Zare Javid et al. (2014)	E	N6
Yano et al. (1979)	E	N3
Nahum et al. (1966)	E	N3
Ringsdorf et al. (1962)	E	N1
El-Sharkawy et al. (2019)	E	N2
Coates et al. (2017)	E	N1
Chapple et al. (2019)	E	N4
Babei et al. (2018)	E	N6
De Angelis et al. (2020)	E	N6

Note: E= Electronic literature search, N1= not controlled and/or randomized, N2= no general healthy subjects, N3= journal articles, N4=not blinded, N5= no periodontal indices (PD/CAL), N6= missing data.

Supplementary Table S2: Data of the meta-analysis including all studies providing values of difference between baseline and three months follow up: (a) PPD, (b) CAL, (c) BOP

(a)

Study	SMD	95% Conf. Interval		% Weight
Stańdo et al. (2020), Omega-3	1.357	0.557	2.158	24.98
Arora_et al. (2013), Lycopene	0.000	-0.605	0.605	25.15
Chopra et al. (2016), Green tea	7.117	6.119	8.114	24.75
Graziani et al. (2018), Kiwi	-1.629	-2.272	-0.986	25.12
D+L pooled SMD	1.691	-1.440	4.823	100.00

Heterogeneity chi-squared = 217.15 (d.f. = 3), p = 0.000
I-squared (variation in SMD attributable to heterogeneity) = 98.6%
Estimate of between-study variance Tau-squared = 10.053

Test of SMD=0 : z = 1.06; p = 0.290

(b)

Study	SMD	95% Conf. Interval		% Weight
Stańdo et al. (2020), Omega-3	2.715	1.705	3.725	24.80
Arora_et al. (2013), Lycopene	2.709	1.862	3.557	24.98
Chopra et al. (2016), Green tea	5.203	4.433	5.974	25.06
Graziani et al. (2018), Kiwi	-1.714	-2.366	-1.062	25.16
D+L pooled SMD	2.223	-1.008	5.454	100.00

Heterogeneity chi-squared = 194.04 (d.f. = 3), p = 0.000
I-squared (variation in SMD attributable to heterogeneity) = 98.5%
Estimate of between-study variance Tau-squared = 10.692

Test of SMD=0 : z = 1.35; p = 0.178

(c)

Study	SMD	95% Conf. Interval		% Weight
Stańdo et al. (2020), Omega-3	-0.637	-1.374	0.099	33.53
Arora_et al. (2013), Lycopene	3.236	2.304	4.168	33.29
Graziani et al. (2018), Kiwi	-4.183	-5.187	-3.180	33.18
D+L pooled SMD	-0.525	-4.372	3.323	100.00

Heterogeneity chi-squared = 113.44 (d.f. = 2), p = 0.000
I-squared (variation in SMD attributable to heterogeneity) = 98.2%
Estimate of between-study variance Tau-squared = 11.352

Test of SMD=0 : z = 0.27; p = 0.789

Supplementary Figure S1: Forest plots for the outcomes of (**a**) PPD, (**b**) CAL and (**c**) BOP.

(a)

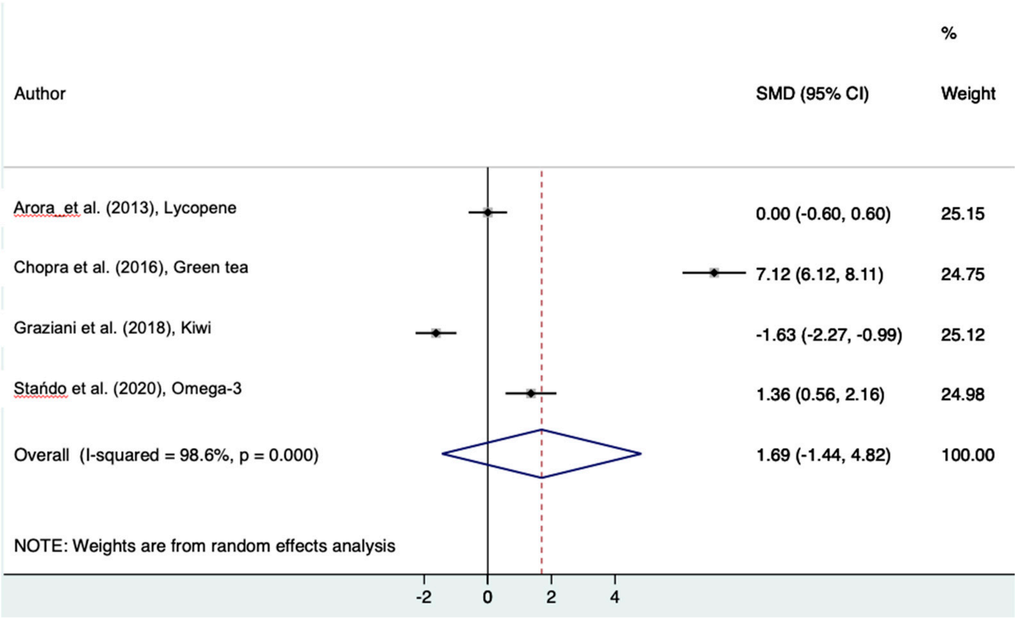

(b)

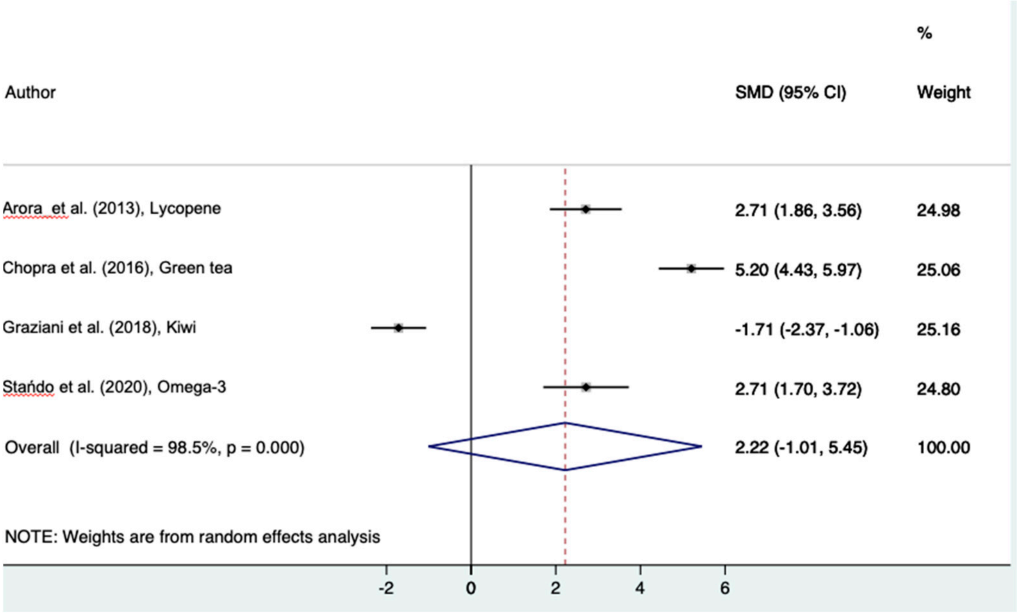

(c)

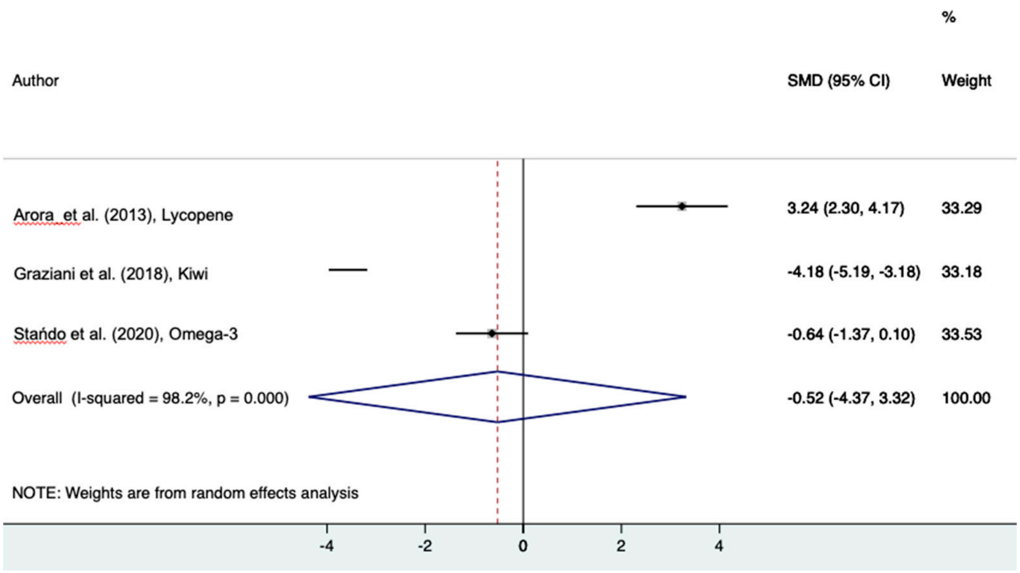

%
Author
SMD (95% CI)
Weight
Arora et al. (2013), Lycopene
3.24 (2.30, 4.17)
33.29
Graziani et al. (2018), Kiwi
-4.18 (-5.19, -3.18)
33.18
Stańdo et al. (2020), Omega-3
-0.64 (-1.37, 0.10)
33.53
Overall (I-squared = 98.2%, p = 0.000)
-0.52 (-4.37, 3.32)
100.00
NOTE: Weights are from random effects analysis
-4
-2
0
2
4

Supplementary Table S3: Comparison of supplemented micronutrients and food intake possibilities; Source: https://fdc.nal.usda.gov/

**Study**	**Intervention (daily)**	**foods (daily)**
**Arora et al. (2013)**	8 mg Lycopene,<br>10,000 IU vitamin A,<br>40 IU ß-tocopherol,<br>200 mg vitamin C,<br>109.8 mg zinc sulfate monohydrate, equivalent to 24.96 mg zinc<br>280 µg selenium dioxide	11 g tomato paste or ripe vine tomato (approx. 115 g)<br>120 g carrots (provitamin A) or 125 g liver<br>135 g raw chicory<br>105 g red bell pepper, raw<br>220 g beef or 400 g pumpkin seeds<br>5 Brazil nuts (approx. 20 g)
**Babaei et al. (2018)**	2 g chicory extract: phenols (96.4 mg/g) and flavonoids (17.4 mg/g)	11 g blueberries
**Kecili et al. (2019)**	400 µg of folate	200g oat flakes<br>100g spinach<br>100g broccoli
**Nafade et al. (2022)**	Oolong tea (appr. 99.32 mg polyphenols)	

**Chapple et al. (2012)**	7.5 mg ß-carotene, 46 mg vitamin E, 200 mg vitamin C 400 µg folic acid + polyphenols (no information about the amount)<br>or<br>7.5 mg ß-carotene, 66 mg vitamin E, 222 mg vitamin C and 640 µg folic acid + polyphenols (quantity not specified).	88 g sweet potatoes, 31 g wheat germ oil, 215 g kiwi fruit.<br>276 g lamb's lettuce + polyphenols from e.g. green tea<br><br>88 g sweet potatoes, 45 g wheat germ oil, 239 g kiwi<br>441 g lamb's lettuce + polyphenols from e.g. green tea
**Chopra et al. (2016)**	Green tea (40-150 mg polyphenols and flavonoids)	-
**Graziani et al. (2017)**	2 kiwi fruits, approx. 140 mg vitamin C	-
**Deore et al. (2014)**	300 mg tablet with omega-3 fatty acids<br>(180 mg EPA and 120 mg DHA)	10 g herring, alternatively algae oil depending on the<br>Omega-3 concentration
**Keskiner et al. (2017)**	6.25 mg EPA and 19.19 mg DHA	0.85 g herring, alternatively algae oil depending on the<br>Omega-3 concentration
**Martinez et al. (2014)**	900 mg tablet with omega-3 fatty acids<br>(540 mg EPA and 360 mg DHA)	30 g herring, alternatively algae oil depending on the<br>Omega-3 concentration
**Gao et al. (2020)**	2000 IU vitamin D3 corresponds to 50 µg or<br>1000 IU vitamin D3 equivalent to 25 µg	200 g herring<br>100 g herring
**Peric et al. (2020)**	Approx. 3570 IU vitamin D3 (25,000 IU / week) corresponds to 94 µg	376 g herring
**Stańdo et al. (2020)**	2.6g EPA, 1.8g DHA, 1.4g alkylglycerol, 1.4g squalene, 240µg vitamin A, 2µg vitamin D3	147 g herring,<br>18g carrots (provitamin A) or 18.9g Liver
**Singh et al. (2014)**	200 mg vitamin E	150 g almonds + 60g weat germ oil + 100 g peanuts + 100 g spinach
